# circPTP4A2-miR-330-5p-PDK2 Signaling Facilitates In Vivo Survival of HuMSCs on SF-SIS Scaffolds and Improves the Repair of Damaged Endometrium

**DOI:** 10.1155/2022/2818433

**Published:** 2022-05-06

**Authors:** Yuanyuan Zheng, Linhao Li, Xuewei Bi, Ruyue Xue

**Affiliations:** ^1^National Engineering Laboratory for Internet Medical Systems and Applications, The First Affiliated Hospital of Zhengzhou University, Zhengzhou University, Zhengzhou, Henan, China; ^2^Key Laboratory of Biomechanics and Mechanobiology, Ministry of Education, Beijing Advanced Innovation Center for Biomedical Engineering, School of Biological Science and Medical Engineering, Beihang University, Beijing, China

## Abstract

**Background:**

Human umbilical cord mesenchymal stem cells- (HuMSCs-) based therapy has shown promising results in the treatment of intrauterine adhesions (IUA). In this study, we aimed to construct a HuMSCs-seeded silk fibroin small-intestinal submucosa (SF-SIS) scaffold and evaluate its ability to repair the damaged endometrium in an IUA mouse model.

**Methods:**

To identify the functional effect of HuMSCs-SF-SIS scaffolds on the repair of damaged endometrium, a mouse IUA model was established. Uterine morphology and fibrosis were evaluated by hematoxylin-eosin staining and Masson staining. CircRNA sequencing, real-time PCR, and RNA fluorescence in situ hybridization were used to screen and verify the potential circRNAs involved in the repair of damaged endometrium by HuMSCs. Real-time integrated cellular measurement of oxygen consumption rate was performed using the Seahorse XF24 Extracellular Flux Analyzer. The potential downstream miRNAs and proteins of circRNAs were analyzed by dual-luciferase reporter assay and western blot.

**Results:**

HuMSCs-SF-SIS not only increased the number of glands but also reduced the ulcer area in the IUA model. circPTP4A2 was elevated in the HuMSCs seeded on the SF-SIS scaffolds and was targeted by miR-330-5p-PDK2. It also stabilized the mitochondrial metabolism of HuMSCs. Moreover, miR-330-5p was found to inhibit PDK2 expression through the 3′ UTR target region. A rescue experiment further showed that circPTP4A2-miR-330-5p-PDK2 signaling was critical to HuMSCs-SF-SIS in decreasing the fibrosis area and increasing the number of glands in the IUA model.

**Conclusion:**

We demonstrated that circPTP4A2 was elevated in HuMSCs-seeded on SF-SIS scaffolds and stabilized the mitochondrial metabolism through miR-330-5p-PDK2 signaling, which contributes to endometrial repair progression. These findings demonstrate that HuMSCs-seeded SF-SIS scaffolds have potential for the treatment of IUA.

## 1. Introduction

Embryo implantation failure caused by endometrial damage is the main factor affecting the success rate of assisted reproduction. Cells in the functional layer of the endometrium in a normal cycle are apoptotic: they shed and release signals to activate endometrial stem cells (ESCs), promoting angiogenesis and tissue repair. However, after damage, the endometrium is deficient in blood vessels and has few glands, leading to thinning of the uterine lining (atrophy) [1, 2]. Intrauterine adhesions (IUA), also called Asherman's syndrome, are caused by damage to the basal lining of the endometrium caused by mechanical injury or infection, resulting in endometrial fibrosis, uterine obstruction, menstrual abnormalities, infertility, and implantation failure [3, 4]. Presently, hysteroscopic adhesiolysis is performed clinically, and antiadhesion drugs and IUD are administered simultaneously [5]. Several therapeutic agents have been used to improve endometrial regrowth [6]. However, due to the loss and functional destruction of endometrial basal stem cells, the therapeutic effect is not ideal, and a normally functional endometrial environment cannot be provided.

Mesenchymal stem cells (MSCs) can differentiate into endometrial epithelial and mesenchymal cells after induction, suggesting that MSCs have biological functions similar to those of basement ESCs and may be involved in the repair of the endometrial structure, improving its function [7]. However, multiple challenges persist in the treatment of endometrial damage by exogenous mesenchymal stem cells, such as differences in the cell carrier and the intrauterine environment [8]. Generally, human umbilical cord MSCs (HuMSCs) are superior to bone marrow MSCs (BM-MSCs) in terms of cell content and proliferative capacity, and they have a lower immunogenicity than BM-MSCs [9, 10]. In addition, HuMSCs have attracted increasing attention because they can be conveniently obtained without ethical concerns and have other advantages [11]. Studies using in vivo HuMSC transplantation experiments to observe endometrial reconstruction two months after the detection of transplantation showed a thickening of the damaged endometrial tissue, a reduction in the area affected by fibrosis, and a similar repair period. It is speculated that HuMSCs secreted via paracrine factors have anti-inflammatory effects, facilitate the repair of the endometrium, and maintain cell function and angiogenesis in the microenvironment [12]. However, the molecular mechanism underlying the effect of HuMSCs on the repair and regeneration of the endometrium remains unclear.

Recently, a great deal of focus has been placed on the application of tissue-engineered artificial reconstruction materials for tissue repair and regeneration. Small-intestinal submucosa (SIS) is a cell-free, allogeneic, and collagen-matrix material with good histocompatibility and biomechanical properties and with no immunogenicity or toxicity [13]. It is a natural biodegradable biological material [14–16]. The submucosa of the small intestine is mainly composed of type I collagen isotype and is rich in fibronectin and growth factors, among which aplasia collagen and fibronectin specifically bind to the cell membrane and activate the cell conduction pathway to facilitate cell adhesion [17]. As a tissue-engineered material, SIS has been widely used in the research of cartilage, tendon, bladder, and cardiovascular regeneration, as well as other fields; however, a few studies have been reported on their role in the repair of injuries of the endometrium and the underlying molecular mechanism [18, 19]. Silk fibroin (SF) has good mechanical properties, controllable biodegradation, and excellent biocompatibility, and it has been widely used in the field of biomedicine [20, 21]. The SIS membrane is a material coated with SF layers, which can adjust the diffusion rate of drugs or biomolecules and improve mechanical and structural stability.

In this study, we constructed HuMSCs-seeded SIS scaffolds and evaluated their impact on the repair of damaged endometrium in an IUA mouse model. Moreover, we studied the potential mechanism of HuMSCs in repairing the endometrium.

## 2. Materials and Method

### 2.1. Preparation and Characterization of SF-SIS Scaffolds

The SF-SIS scaffolds used in this study were donated from Dr. Li (The Key Laboratory for Biomechanics and Mechanobiology of Ministry of Education, School of Biological Science and Medical Engineering, Beihang University, Beijing, China). The SF-SIS scaffolds were prepared and characterized as previously described [20].

### 2.2. Characterization and Culture of HuMSCs

HuMSCs were purchased from PromoCell (Miaotong (Shanghai) Biological Science and Technology Co., Ltd., Shanghai, China). P4-P5 frozen HuMSCs were freshly inoculated in 100 mm culture dishes (1 × 10^6^ cells per dish) supplemented with 10% (*v*/*v*) fetal bovine serum (FBS, Gibco, USA), penicillin (100 U/ml, Gibco), and *Streptomyces* (100 mg/ml, Gibco). Briefly, the phenotype of HuMSCs was identified by FACS specificity (CD34, CD45, CD29, CD90, CD105, and HLA-DR). The osteogenic and adipogenic abilities of mesenchymal stem cells were determined by the Cyagen Osteogenic stimulation kit (Cyagen, Guangzhou, China) and the Cyagen Adipogenic Differentiation Kit (Cyagen, Guangzhou, China).

### 2.3. Osteogenic and Adipogenic Differentiation of HuMSCs

According to the manufacturer's instructions (RASMX-90021 and RASMX-90031, Cyagen, Guangzhou, China), the ability of cells to differentiate into osteoblasts or adipocytes was characterized using commercial osteogenic and adipogenic induction kits. Osteoblasts were stained with alizarin red S and adipocytes with oil red O.

### 2.4. Animal IUA Model

All animal procedures were approved by the first affiliated hospital of Zhengzhou University, Institutional Animal Care and Use Committee, and were carried out in accordance with the National Research Council Laboratory Animal Care and Use Guidelines. Six-week-old BALB/c mice were obtained from Shanghai SLAC Animal Center and allowed to acclimatize to the new environment for one week. To establish an IUA model, each uterus was mechanically damaged during pregnancy. Mice were anesthetized using intraperitoneal injection of sodium pentobarbital. Each uterus was excised at the midline below the abdomen. A size 7 needle was inserted into the left and right uterine junctions and carefully moved back and forth until the uterine congestion was visible to the naked eye. Then, the abdominal cavity was closed. The operation was performed under aseptic conditions. The control group did not undergo surgery. To analyze the role of circPTP4A2-miR-330-5p-PDK2 signaling in the progress of the repair of the endometrium by HuMSCs-SF-SIS, we transfected miR-330-5p mimic, PDK2 siRNA, or pcDNA3-PDK2 overexpression vector in HuMSCs-SF-SIS.

### 2.5. Histological Analysis

Uterine specimens were collected 8 days after surgery. The samples were fixed with 4% paraformaldehyde, dehydrated, removed with xylene, and finally, embedded with paraffin. The embedded tissue sections were 5 *μ*m thick. The uterine morphology and structure were evaluated by hematoxylin-eosin (H&E) staining. Masson staining was performed to evaluate the fibrosis according to the manufacturer's instructions (Yeasen, Shanghai, China). Five high-magnification fields were selected for each H&E-stained section, and the number of glands in each field of view was counted and averaged. Five high-magnification fields were selected for each Masson-stained slice, and the fibrosis area ratio was calculated as follows: total area of endometrial fibrosis per field/the sum area of endometrial stroma. The rate was automatically averaged using the ImageJ software (Media Cybernetics, Inc., MD, USA).

### 2.6. circRNA Sequencing

Total RNAs were extracted with TRIzol (Invitrogen, Carlsbad, CA, USA). Sequencing libraries were generated and sequenced by Sango Biotechnology (Shanghai, China). A total amount of 5 *μ*g RNA per sample was used. The libraries were subjected to paired-end sequencing with pair-end 150 bp reading length on an Illumina HiSeq sequencer (Illumina, San Diego, CA, USA).

### 2.7. Real-Time PCR

Total RNA was extracted with TRIzol reagent (TaKaRa), and 500 ng total RNA was transcribed into cDNA using PrimeScript RT Master Mix (TAKARA, Dalian, China). Subsequently, SYBR Premix Ex Taq II Kit (TaKaRa) was used to detect the expression of circRNAs and mRNA by RT-qPCR, which was normalized to endogenous control GAPDH expression. Folding changes were calculated using 2^−*ΔΔ*CT^ method.

### 2.8. RNA Fluorescence In Situ Hybridization

RNA fluorescence in situ hybridization was performed using a fluorescence in situ hybridization kit (RiboBio, Guangzhou, China) in accordance with the manufacturer's guidelines. Cy3-labeled circPTP4A2 probe (RiboBio, Guangzhou, China) was detected with fluorescence in situ hybridization kit and then observed with LSM800 confocal microscopy (Zeiss, Germany).

### 2.9. Measurement of Mitochondrial Metabolism

Analyses of ATP content and synthase activity were performed using quantification kits (Sango Biotech, Shanghai, China) in accordance with the manufacturer's guidelines. Real time integrated cellular oxygen consumption rate (OCR) was measured using the Seahorse XF24 Extracellular Flux Analyzer (Seahorse Bioscience, North Billerica, MA, USA) as previously described. In brief, HuMSCs were treated with 10 *μ*g/mL curcumin for 12 h, and 10^4^ cells were plated onto the seahorse customized cell plates. After the probes were calibrated, the OCR was detected with sequential injection of the following compounds which regulate mitochondrial respiration: oligomycin (ATP synthase inhibitor; 1 *μ*M), FCCP (uncoupler; 1 *μ*M), rotenone (complex I inhibitor; 1 *μ*M), and antimycin A (complex III inhibitor; 1 *μ*M).

### 2.10. Dual-Luciferase Reporter Assays

The 293T cells were incubated in 24-well plates with 2 × 10^4^ cells per well. These cells were then transfected with psiCheck2-circPTP4A2-WT or psiCheck2-circPTP4A2-Mut, miR-330-5p mimics, or miR-NC. After 48 h, the cells were lysed with a passive lysis buffer (Promega, Madison, WI, USA), and the dual luciferase reporting assay (Promega) was used to calculate the relative luciferase activity by normalized firefly luminescence to Renilla luminescence.

### 2.11. AGO2 RNA-Immunoprecipitation Assay

AGO2 RNA-immunoprecipitation (RIP) assay was conducted to determine the enrichment of miR-330-5p and PDK2 3′ UTR in the RNA induced silencing complex. RIP assay AGO2 from HuMSCs was conducted. Real-time PCR analysis of whole-cell lysates and immunoprecipitates with IgG or AGO2 antibodies was performed.

### 2.12. RNA Pull-Down Assay

A total of 1 × 10^7^ HuMSCs were harvested, lysed, and sonicated. The miR-330-5p WT and mutant probes were used for incubation with C-1 magnetic beads (Life Technologies) at 25°C for 2 h to generate probe-coated beads. Cell lysate with miR-330-5p WT and mutant probes or oligo probe was incubated at 4°C for one night. After washing with wash buffer, the RNA mix bound to the beads was eluted and extracted with the RNeasy Mini Kit (QIAGEN) for real-time PCR of GAPDH or circPTP4A2.

### 2.13. Western Blotting

The HuMSCs were lysed with RIPA lysis buffer. The same amount of protein was then broken down by SDS-PAGE analysis and electrically transferred to PVDF membranes (Millipore, Schwalbach, Germany), which were then sealed with 5% skimmed milk powder and incubated overnight with primary antibody at 4°C. The primary antibodies used were anti-PDK2 (Cell Signaling Technology) and anti-GAPDH (Kanchen Biotech). The membrane was then incubated with HRB-conjugated secondary antibodies at room temperature for 1 h, and the imprinting was observed using an enhanced chemiluminescence kit (Pierce, Waltham, MA, USA).

### 2.14. Statistical Analysis

Data were expressed as the means ± SEM. For comparisons of two groups, two-tailed *t*-test (unpaired) was used. For multiple comparisons, ANOVA followed by the post hoc Bonferroni test was taken with the GraphPad Prism® version 9.0 software (GraphPad Software, Inc., La Jolla, CA, USA).

## 3. Results

### 3.1. Culture and Characterization of HuMSCs

**Figure 1:**
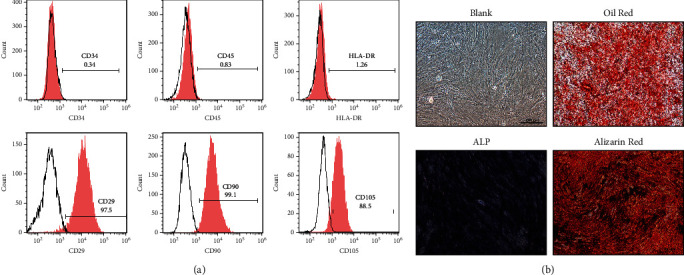
Culture and characterization of HuMSCs. (a) The cell morphology of the cultured HuMSCs. (b) The cell markers were analyzed by FACS. *N* = 3.

The typical morphology of HuMSCs is similar to that of spindle-shaped fibroblasts, which grow in a tightly packed vortex pattern (Figure 1(a)). The HuMSCs were successfully differentiated into osteoblasts and adipocytes in vitro (Figure 1(b)), indicating that this cell population is a pluripotent mesenchymal stromal cell. Furthermore, FACS results showed that most cells were negative for hematopoietic markers after 7 days in culture, including CD34 (0.34%), CD45 (0.83%), and HLA-DR (1.26%), and highly positive for CD29 (97.5%), CD90 (99.1%), and CD105 (88.5%) (Figure 1(b)).

### 3.2. HuMSCs-SF-SIS Reduces Fibrotic Area and Increases the Number of Glands in the lUA Model

**Figure 2:**
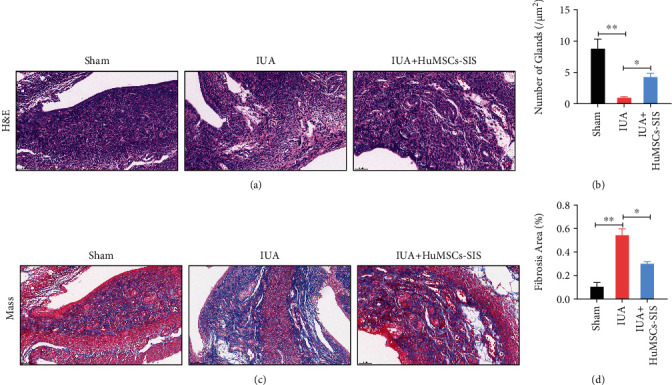
HuMSCs-SF-SIS increased the number of glands and reduced the area of fibrosis in the lUA model. (a) The number of endometrial glands and the lumen of neonates were analyzed using HE staining. (b) Statistical analysis was performed to evaluate the number of endometrial glands. (c) The extent of fibrosis was assessed with Masson staining. (d) The fibrotic area of endometrium was assessed with the ImageJ software. *N* = 3. ^∗^*P* < 0.05 relative to the indicated group.

To identify the functional effect of HuMSCs-SF-SIS scaffolds on the repair of damaged endometrium, a mouse lUA model was established. The HuMSCs-SF-SIS scaffolds were transplanted after the damage to the endometrium was reduced. After 4 weeks, uterine tissue was collected for modeling. HE staining results revealed that the shape of the uterine cavity in the pseudopod group was irregular, that columnar epithelial cells covered the uterus and gland cavities, that the epithelial cell structure was complete, and that stromal glands were abundant and oval. Connective tissue fragments were found in the uterine cavity in the IUA model group, the number of glandular blood vessels was significantly reduced, and the connective tissue was congested one week after the damage. The number of endometrial glands in the HuMSCs-SF-SIS-transplanted group increased, and the lumen of neonates was not completely covered by the monolayer columnar epithelium (Figure 2(a) , *P* < 0.05). The number of endometrial glands in the model group and HuMSCs-SF-SIS transplantation group was lower than those in the sham-operated group (Figure 2(b), *P* < 0.05), and that in the model group was significantly lower than that in the sham-operated group (*P* < 0.05). By contrast, the number of endometrial glands in the HuMSCs-SF-SIS transplantation group was significantly higher than that in the IUA model group (Figure 2(b), *P* < 0.05). Endometrial adhesions are characterized by fibrosis; in the study, we used Masson staining (Figure 2(c)) to assess the degree of fibrosis. A significant increase in the area of endometrial fibrosis was observed in IUA model mice compared with that in the sham group (Figure 2(d), *P* < 0.05), but HuMSCs-SF-SIS transplantation resulted in a significant decrease in fibrosis (Figure 2(d), *P* < 0.05). In conclusion, transplantation of HuMSCs-SF-SIS not only increased the number of endometrial glands but also repaired the injured endometrium.

### 3.3. The Expression of circPTP4A2 Was Significantly Elevated in HuMSCs Cultured on SF-SIS Scaffolds

**Figure 3:**
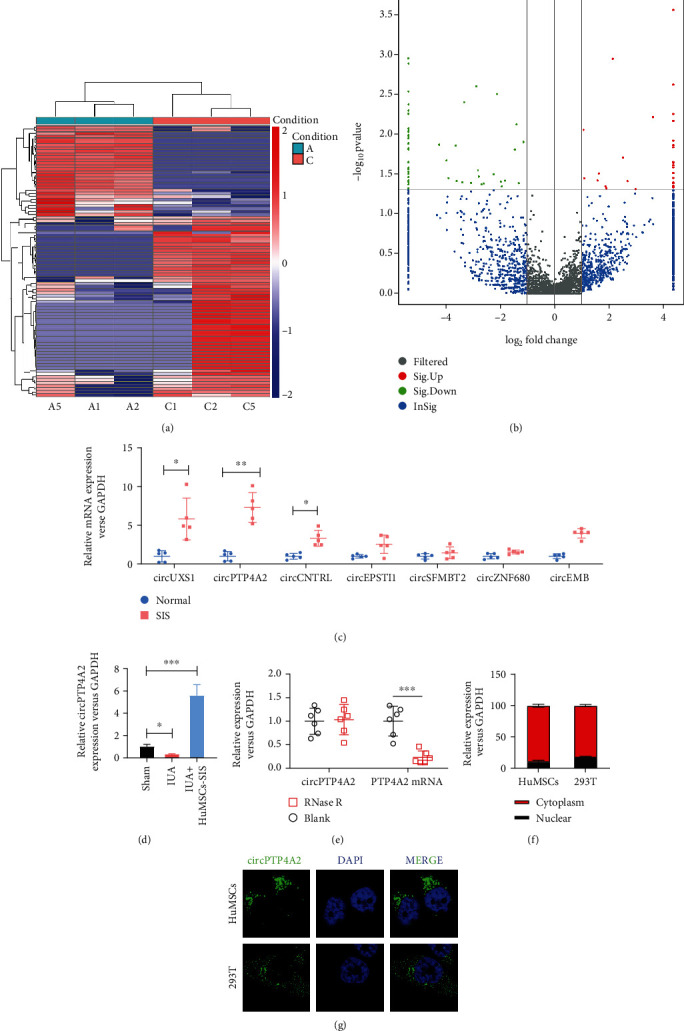
The expression of circPTP4A2 is significantly elevated in HuMSCs cultured on SF-SIS scaffolds. (a) The profile of circRNA from normal HuMSCs and HuMSCs cultured on the SF-SIS scaffolds was analyzed by RNA-seq of ribosomal RNA-depleted total RNA. (b) The differential expression of total circRNAs was directly revealed by volcanic eruption-type clustering analysis. (c) Several significantly upregulated circRNAs (circUXSI, circPTP4A2, circCNTRL, circEPSTM, circSFMBT2, circZNF680, and circEMB) were verified via real-time PCR. (d) The expression level of circPTP4A2 in the different animal models was analyzed via real-time PCR. (e) CircPTP4A2 was identified using real-time PCR, indicating resistance between circPTP4A2 and RNase R. RNase R treatment significantly reduced PTP4A2 mRNA. (f) Subfractional real-time PCR and (g) fluorescence in situ hybridization (FISH) assay were used to analyze the subcellular location of circPTP4A2 in HuMSCs. *N* = 3, *P* < 0.05 relative to the indicated group.

To explore whether circRNA is involved in endometrial repair, we first performed RNA-seq analysis of total RNA from ribosomal RNA of normal HuMSCs and HuMSCs cultured on SF-SIS scaffolds (Figure 3(a)). A total of 54 circRNAs were significantly downregulated, and 27 circRNAs were upregulated in HuMSCs cultured on the SF-SIS scaffolds (filtered by FC (fold change) > 2 and *P* < 0.05). Differential expression of circRNAs was directly displayed by volcano clustering analysis (Figure 3(b)). We then chose several significantly upregulated circRNAs (circUXSI, circPTP4A2, circCNTRL, circEPSTII, circSFMBT2, circZNF680, and circEMB) to verify their expression via real-time PCR (Figure 3(c)). We concluded that circPTP4A2 was upregulated in HuMSCs cultured on the SF-SIS scaffolds (Figure 3(c)) and in HuMSCs-SF-SIS-transplanted endometrial tissues in the IUA model. In a follow-up study, we found that circPTP4A2 resisted degradation by RNase R and confirmed that PTP4A2 mRNA showed a significant reduction after RNase R treatment (Figure 3(e)). Moreover, subfractional real-time PCR (Figure 3(f)) and fluorescence in situ hybridization assays (Figure 3(g)) indicated that circPTP4A2 was mainly present in the cytoplasm.

### 3.4. CircPTP4A2 Facilitates the Mitochondrial Metabolism of HuMSCs under Hypoxia Conditions

**Figure 4:**
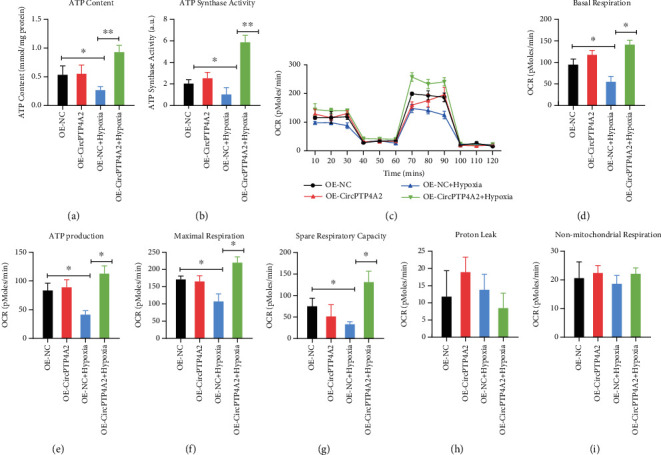
CircPTP4A2 facilitates the mitochondrial metabolism of HuMSCs under hypoxia condition. (a) The effects of circPTP4A2 on ATP content and (b) ATP synthase activity were analyzed using kits. (c) The cell oxygen consumption rate (OCR) of HuMSCs was analyzed with the seahorse XF24 Extracellular Flux Analyzer. (d) Basal respiration, (e) ATP production, (f) maximal respiration, (g) spare respiratory capacity, (h) proton leak, and (i) nonmitochondrial respiration were analyzed. *N* = 3. ^∗^*P* < 0.05 relative to the indicated group.

Mitochondria are essential in the cellular biochemistry of most eukaryotic cells, producing nearly 95% of cellular ATP through oxidative phosphorylation and thereby controlling cell death or survival under hypoxic conditions, such as in the transplanted SF-SIS scaffolds. Considering the critical role of mitochondrial metabolism, we tested whether circPTP4A2 facilitates the biological functions of HuMSCs via the regulation of mitochondrial metabolism. For the purpose of hypothesis testing, we first determined the effects of circPTP4A2 on ATP content (Figure 4(a)) and ATP synthase activity (Figure 4(b)). We found that the ATP content and ATP synthase activity were dramatically decreased in hypoxic HuMSCs but significantly increased with the overexpression of circPTP4A2. Using the seahorse XF24 extracellular flux analyzer, we also analyzed the cell OCR (Figure 4(c)), revealing reduced oxidative phosphorylation in HuMSCs. We then assessed the mitochondrial functions, including basal respiration (Figure 4(d)), maximal respiration (Figure 4(f)), ATP production (Figure 4(e)), spin respiratory capacity (Figure 4(g)), proton leak (Figure 4(h)), and nonmitochondrial respiration (Figure 4(i)). Hypoxia treatment dramatically reduced OCR during basal respiration, spare respiratory capacity, maximal respiration, and ATP production in HuMSCs. In contrast, overexpression of circPTP4A2 significantly attenuated the inhibitory effect of hypoxia on ATP content, ATP synthase activity, and cell OCR. These data indicate that circPTP4A2 could facilitate the mitochondrial metabolism of HuMSCs under hypoxic conditions.

### 3.5. CircPTP4A2 Is Targeted by miR-330-5p in HuMSC Cells

**Figure 5:**
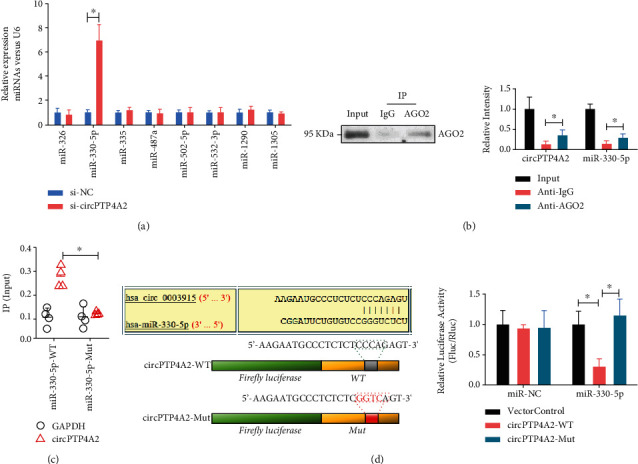
CircPTP4A2 is targeted by miR-330-5p in HuMSC cells. (a) The levels of miRNAs (miR-487a, miR-330-5p, miR-1290, miR-326, miR421, miR-502-5p, miR-532-3p, miR-335, and miR-1305) in HuMSCs with or without circPTP4A2 knockout were analyzed using real-time PCR. (b) Ago2 coimmunoprecipitation assay and (c) miR-330-5p RNA pull-down were used to evaluate the interaction between emiR-330-5p and circPTP4A2. (d) Dual-luciferase reporter assay was used to evaluate the effect of miR-30-5p on circPTP4A2 in HuMSCs. *N* = 3. ^∗^*P* < 0.05 relative to the indicated group.

To explore whether circPTP4A2 can function as a “miRNA sponge” in HuMSCs, we selected several potential miRNAs (miR-326, miR-487a, miR-335, miR-532-3p, miR-421, miR-502-5p, miR-330-5p, miR-1290, and miR-1305) from the starBase 2.0 database. After circPTP4A2 knockout, we found an increased level of miR-330-5p (Figure 5(a)) in HuMSCs. Enrichment of miR-330-5p with circPTP4A2 was observed by Ago2 coimmunoprecipitation assay (Figure 5(b)) and miR-330-5p RNA pull-down assay (Figure 5(c)). To further verify that miR-330-5p targets circPTP4A2, we performed a luciferase reporter gene test. The results showed that miR-330-5p mimics significantly reduced the luciferase activity of HuMSCs transfected with wild-type circPTP4A2 but failed to reduce the luciferase activity of the mutant circPTP4A2-transfected HuMSCs (Figure 5(d)). These results suggest that circPTP4A2 is targeted by miR-330-5p in HuMSCs.

### 3.6. MiR-330-5p Overexpression Impaired the circPTP4A2-Enhanced Mitochondrial Metabolism in Hypoxia-Treated HuMSCs

**Figure 6:**
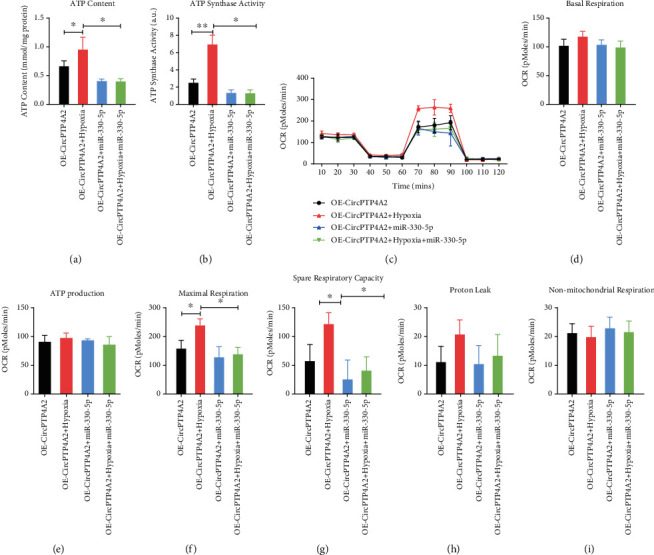
MiR-330-5p overexpression impairs the circPTP4A2-enhanced mitochondrial metabolism in hypoxia-treated HuMSCs. (a) The rescue effects of miR-330-5p on the circPTP4A2-enhanced ATP content and (b) ATP synthase activity were analyzed using kits. (c) The rescue effects of miR-330-5p on the circPTP4A2-enhanced cell oxygen consumption rate (OCR) of HuMSCs were analyzed with the seahorse XF24 Extracellular Flux Analyzer. (d) Basal respiration, (e) ATP production, (f) maximal respiration, (g) spare respiratory capacity, (h) proton leak, and (i) nonmitochondrial respiration were analyzed. *N* = 3. ^∗^*P* < 0.05 relative to the indicated group.

To determine whether miR-330-5p is critical to circPTP4A2-enhanced mitochondrial metabolism in hypoxia-treated HuMSCs, we transfected the miR-330-5p mimic into hypoxia-treated HuMSCs with or without circPTP4A2 overexpression. The miR-330-5p mimic dramatically reduced ATP content (Figure 6(a)) and ATP synthase activity (Figure 6(b)). Moreover, the miR-330-5p mimic reduced basal respiration, spare respiratory capacity, ATP production, and maximal respiration in HuMSCs (Figure 6(c)). Notably, circPTP4A2 overexpression failed to increase basal respiration (Figure 6(d)), ATP production (Figure 6(e)), maximal respiration (Figure 6(f)), and spare respiratory capacity (Figure 6(g)) in HuMSCs transfected with miR-330-5p mimic. These results show that miR-330-5p overexpression impaired circPTP4A2-enhanced mitochondrial metabolism in hypoxia -treated HuMSCs.

### 3.7. MiR-330-5p Inhibits PDK2 Expression through the 3′ UTR Target Region

**Figure 7:**
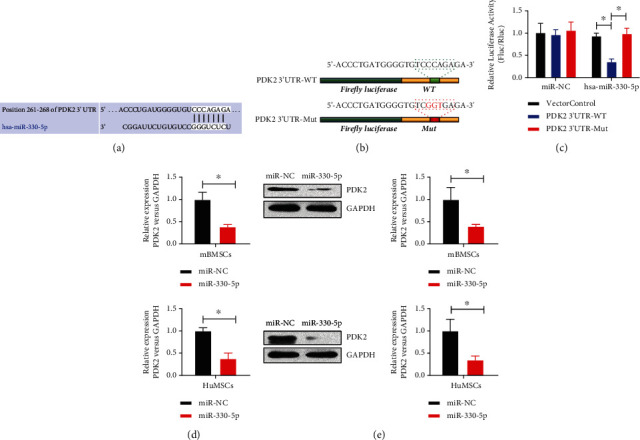
MiR-330-5p suppresses the expression of PDK2 via the 3′ UTR target region. (a–c) Dual-luciferase reporter assay was used to evaluate the effect of miR-30-5p on PDK2 mRNA 3′ UTR in HuMSCs. (d) The mRNA and (e) protein levels of PDK2 in mouse umbilical MSCs and HuMSCs were analyzed using real-time PCR and western blot. *N* = 3. ^∗^*P* < 0.05 relative to the indicated group.

To further explore the underlying mechanism of the effect of the circPTP4A2/miR-330-5p axis on mitochondrial metabolism in hypoxia-treated HuMSCs, we screened the potential targets of miR-330-5p using the TargetScan and starBase databases. Dual luciferase assay revealed that the 3′ UTR mRNA of the mitochondrial metabolism regulator PDK2 was directly targeted by miR-330-5p (Figures 7(a)–7(d)). In addition, PDK2 mRNA (Figure 7(b)) and protein (Figure 7(c)) levels were significantly reduced by miR-330-5p mimics in mouse umbilical cord MSCs and HuMSCs. These results demonstrate that miR-330-5p may regulate mitochondrial metabolism by suppressing the expression of PDK2.

### 3.8. CircPTP4A2-miR-330-5p-PDK2 Signaling Is Critical to HuMSCs-SF-SIS-Mediated Decrease in the Fibrosis Area and Increase of the Number of Glands in the IUA Model

**Figure 8:**
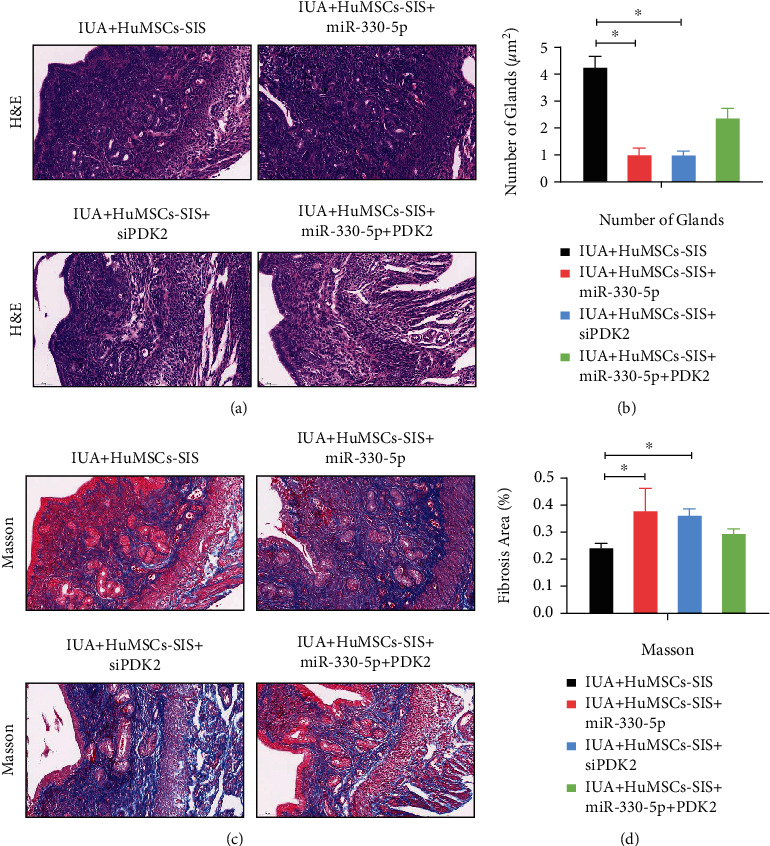
CircPTP4A2-miR-330-5p-PDK2 signaling is critical to the HuMSCs-SF-SIS-mediated increase in the number of glands and decrease in fibrosis area in the IUA model. (a) The number of endometrial glands and the lumen of neonates were analyzed with HE staining. (b) Statistical analysis was performed to evaluate the number of endometrial glands. (c) The degree of fibrosis was assessed using Masson staining. (d) The fibrotic area of endometrium was measured using the ImageJ software. *N* = 3. ^∗^*P* < 0.05 relative to the indicated group.

To confirm the critical role of circPTP4A2-miR-330-5p-PDK2 signaling in the progress of the repair of the endometrium by HuMSCs-SF-SIS in the IUA model, we altered the levels of miR-330-5p and PDK2 in HuMSCs-SF-SIS. As shown in Figures 8(a) and 8(b), the number of endometrial glands in the HuMSCs-SF-SIS-transplanted group was significantly reduced by miR-330-5p and PDK2 knockdown (Figures 8(a) and 8(b), *P* < 0.05). However, PDK2 overexpression significantly enhanced the endometrial glands in the miR-330-5p mimic-transfected HuMSCs-SF-SIS-transplanted group (Figures 8(a) and 8(b), *P* < 0.05). Consistently, Masson staining (Figures 8(c) and 8(d)) results showed that the area of fibrosis in the endometrium of IUA model mice was significantly increased in the miR-330-5p or PDK2 knockdown group, but PDK2 led to a remarkable reduction in fibrosis (Figure 8(d), *P* < 0.05) in the miR-330-5p mimic-transfected HuMSCs-SF-SIS-transplanted group. These results indicate that circPTP4A2-miR-330-5p-PDK2 signaling is critical to HuMSCs-SF-SIS-mediated increase in the number of glands and decrease in the area of fibrosis in the IUA model.

## 4. Discussion

Presently, 2.8–45.5% of women with IUA have impaired fertility, notably occurring after pregnancy-related dilatation and curettage in more than 90% of cases [5, 6]. Until now, IUA has been treated mainly with both surgical and estrogenic modalities, but the recurrence rate is high, ranging from 20% to 63% [3]. Additionally, there is a high risk of placental implantation. Therefore, it is particularly important to develop safe and feasible treatment options for patients with IUA.

MSCs have now been widely used for tissue repair. Gargett et al. proposed that endometrial tissue can be reconstructed in LUA patients using endometrial mesenchymal stem cells [1]. Nagori et al. showed that BM-MSCs transplantation was effective in promoting repair of damaged endometrium in vivo [22], a view shared by Phermthai et al. MSCstransplantation has been reported to be effective in repairing endometrial defects, such as infertility and endometrial hyperplasia [23, 24]. In fibrotic diseases, MSCs play an antifibrotic role; for example, pulmonary fibrosis, renal fibrosis, and hepatic fibrosis can be treated with mesenchymal stem cells [25–29]. The submucosa of the small intestine (SIS) has been widely used in tissue repair and in clinical trials to serve as the extracellular matrix for tissues, such as skin, bone, bladder, ligaments, and the abdominal wall [13]. After SF coating by a single-component LbL assembly, the SIS membrane exhibited good cell compatibility and was not only well-resistant to rapid degradation but also maintained its structural integrity [20]. In this study, we seeded HuMSCs on the surface of the SF-SIS scaffold and were surprised to find an increase in the number of HuMSCs-seeded SF-SIS scaffold glands, as well as a reduction in the fibrotic area of the IUA model. Based on the study data, we conclude that HuMSC-seeded SF-SIS scaffolds may be used for IUA treatment.

CircRNAs are circular noncoding RNAs that are resistant to the digestive action of RNase R. CircRNAs are mainly generated by selective splicing (post splicing) of information exchange between upstream splice acceptors and downstream splice donors [30, 31]. Our comparison of circRNAs with long-stranded noncoding RNAs (lncRNAs) and microRNAs (miRNAs) in mammalian cells revealed that circRNAs are better in terms of stability and conservation [32]. Meanwhile, circRNA interferes with the expression of related genes and with RNA responses, transcribes, and acts as a scaffold or template to assemble or synthesize protein complexes through circRNA sponge action [33]. Recent reports have confirmed the role of several functional circRNAs in regulating tissue regeneration of MSCs. For example, the pluripotency of human embryonic stem cells is maintained by sponge transfection of circBIRC6 with miR-34a and miR-145 [34]. CircHIPK3 has been reported to promote a variety of cancers by absorbing multiple miRNAs through sponge uptake [35]. CircSMARCA5 inhibits glioblastoma pleomorphic cell migration but facilitates prostate cancer cell proliferation [36]. In this study, we found that circPTP4A2 is critical to HuMSCs-SF-SIS-mediated increase in the number of glands and decrease in the fibrosis area in the IUA model by targeting miR-330-5p-PDK2 signaling.

A distinctive feature of eukaryotic cells is the presence of intracellular mitochondria, which play an important role in energy metabolism and apoptosis and are essential in biological longevity [37]. In cells, mitochondria carry out complex biological reactions and contain one of the most complex reactive sensing systems [38]. Recent studies have shown that balancing mitochondrial dynamics, which regulate the fate of stem cells, and morphology is crucial for maintaining tissue homeostasis [39]. Recent studies have found that mitochondrial metabolism can be significantly altered by environmental stimuli [40]. An important feature of the MSC niche is hypoxia, which has been shown over the last decade to have a key role in maintaining stem cell survival, self-replication, and pluripotency [41]. It was found that for the glycolytic pathway, transcription, and synthesis of enzymes increased in hypoxic cells, but synthesis of proteins involved in mitochondrial catabolism decreased [42]. The proliferation, differentiation, and survival of BM-MSCs have been shown to be affected by culture under hypoxic pressure [43]. Cytochrome oxidase, an enzyme located at the end of the mitochondrial respiratory chain, is involved in aerobic synthesis in mammalian cells, mainly using oxygen as a substrate [27]. Under hypoxic conditions, mitochondrial size and average velocity were significantly reduced [44]. In this study, we found that circPTP4A2-miR-330-5p-PDK2 signaling is critical for the stability of mitochondrial metabolism in HuMSCs under hypoxic conditions. Collectively, we constructed HuMSC-seeded SF-SIS scaffolds and evaluated their impact in repairing damaged endometrium in an IUA mouse model. We performed an in-depth study of the underlying mechanisms of endometrial repair progression in HuMSCs, finding that circPTP4A2 was elevated in the HuMSCs seeded on the SF-SIS scaffolds and stabilized mitochondrial metabolism via miR-330-5p-PDK2 signaling. Furthermore, these findings demonstrated that HuMSC-seeded SF-SIS scaffolds have potential for future clinical applications in the treatment of IUA.

## 5. Conclusion

In this study, we demonstrated that circPTP4A2 was elevated in HuMSCs seeded on SF-SIS scaffolds and that it stabilized mitochondrial metabolism through miR-330-5p-PDK2 signaling, which contributed to endometrial repair progression. These findings demonstrate that HuMSC-seeded SF-SIS scaffolds have potential for the treatment of IUA.

## Data Availability

The data that support the findings of this study are available from the corresponding author upon reasonable request.

## Additional Points

*Preprint Statement*. A preprint has previously been published [45].
